# Diminished Returns of Educational Attainment on Hypertension Prevalence among American Indian and Alaska Native Adults: National Health Interview Survey 2023

**DOI:** 10.31586/gjcd.2025.1148

**Published:** 2025-01-23

**Authors:** Shervin Assari, Hossein Zare

**Affiliations:** 1 Department of Internal Medicine, Charles R. Drew University of Medicine and Science, Los Angeles, CA, United States; 2 Department of Family Medicine, Charles R. Drew University of Medicine and Science, Los Angeles, CA, United States; 3 Department of Urban Public Health, Charles R. Drew University of Medicine and Science, Los Angeles, CA, United States; 4 Marginalization-Related Diminished Returns (MDRs) Center, Los Angeles, CA, United States; 5 Department of Health Policy and Management, Johns Hopkins Bloomberg School of Public Health, Baltimore, MD, United States; 6 School of Business, University of Maryland Global Campus (UMGC), Adelphi, MD, United States

**Keywords:** Minorities’ Diminished Returns, Educational Attainment, Hypertension Risk, American Indian and Alaska Native (AIAN) Adults, National Health Interview Survey, Racial Health Disparities, Structural Inequities, Social Determinants of Health, Labor Market Discrimination, Cardiovascular Health, Health Inequalities, Social Stratification, Public Health Interventions, Systemic Racism, Intergenerational Trauma

## Abstract

**Background:**

Research on Minorities’ Diminished Returns (MDRs) consistently reveals that social determinants of health, especially educational attainment, do not yield equal health benefits across racial and ethnic groups in the United States. MDRs suggest that social stratification, segregation, lower education quality, and labor market discrimination contribute to diminished health returns of education among minoritized groups. However, few studies have tested the relevance of MDRs in American Indian and Alaska Native (AIAN) populations compared to non-Hispanic White adults.

**Objectives:**

This study aimed to examine the strength of the inverse association between educational attainment and hypertension prevalence, hypothesizing that the protective effect of education on hypertension risk is reduced among AIAN adults relative to non-Hispanic Whites.

**Methods:**

Using data from the 2023 National Health Interview Survey (NHIS), we analyzed a nationally representative sample of adults aged 18 and older. Logistic regression models examined the association between educational attainment and self-reported hypertension diagnosis, stratified by racial/ethnic group (AIAN vs. non-Hispanic White). Models were adjusted for key covariates, including age, gender, income, and insurance status.

**Results:**

Higher educational attainment was associated with a lower prevalence of hypertension in the combined sample of AIAN and non-Hispanic White adults. However, this protective association was significantly weaker among AIAN adults compared to non-Hispanic White adults, as evidenced by a significant interaction between race and education.

**Conclusion:**

AIAN adults exhibit a higher prevalence of hypertension even at higher levels of educational attainment compared to non-Hispanic White adults, supporting the relevance of MDRs for AIAN populations. This finding underscores the need for public health interventions that address structural barriers and contextual factors unique to AIAN populations. Policies focused solely on educational access may be insufficient to reduce hypertension risk among AIAN adults without addressing broader social and structural inequities.

## 1. Introduction

Hypertension, a significant contributor to stroke, cardiovascular disease, kidney disease, disability, and mortality, [1–5] disproportionately affects American Indian and Alaska Native (AIAN) populations in the United States [6–8]. This elevated risk is linked to a combination of risk factors, including lower socioeconomic status, limited access to nutritious food, higher rates of obesity, and elevated tobacco and substance use [9,10]. While these factors are widely recognized, we know that social determinants, such as educational attainment, influence hypertension risk in AIAN populations [5,11,12].

Education is generally associated with improved health behaviors, greater access to health resources, healthier lifestyles, and increased health literacy, all of which can reduce the risk of hypertension. However, research has shown that the protective effects of socioeconomic resources like education and income do not benefit all racial and ethnic groups equally, leading to higher rates of hypertension or elevated blood pressure in some minoritized groups even with similar educational or income levels. For example, previous studies have documented that Black and Latino individuals experience weaker health benefits from socioeconomic status (SES), including education, when it comes to controlling hypertension and blood pressure.

While these patterns have been documented in Black [13–19] and Latino [20–24] populations, evidence remains sparse for AIAN populations [25–27]. Some studies indicate that AIAN individuals may also experience diminished protective effects of their own or parental socioeconomic resources on health outcomes, though hypertension specifically has not been extensively studied in this context [25–27].

Minorities’ Diminished Returns (MDRs) [28,29] describe this phenomenon, in which marginalized groups do not experience the same protective health effects from socioeconomic resources as their non-Hispanic White counterparts. Structural barriers, systemic discrimination, and historical inequities may erode the protective effects of education on health outcomes, including hypertension, in marginalized, racialized and historically oppressed groups such as AIAN adults [25–27]. Indeed, existing research has shown that AIAN populations face elevated rates of hypertension and cardiovascular disease due to chronic social stressors and limited access to equitable healthcare resources [5,11,12].

This study seeks to address this gap by examining whether higher educational attainment confers the same protective benefit against hypertension prevalence for AIAN adults as it does for non-Hispanic White adults. Using data from the 2023 National Health Interview Survey (NHIS), the latest nationally representative survey of health in the United States, we aim to assess whether higher education is associated with reduced hypertension prevalence and to test if the protective effect of education on hypertension risk is attenuated among AIAN adults, consistent with the MDRs framework [30–34]. Understanding these disparities is critical for developing targeted interventions and informing policy efforts to promote cardiovascular health equity for AIAN communities.

## 2. Methods

### 2.1. National Health Interview Survey (NHIS)

The National Health Interview Survey (NHIS) is the principal source of health data for the civilian, noninstitutionalized U.S. population, conducted by the National Center for Health Statistics (NCHS) since 1957. Authorized by the National Health Survey Act of 1956, NHIS continuously gathers data on health and disability across various demographics and socioeconomic groups, supporting research and policy initiatives within the Department of Health and Human Services and beyond [35].

### 2.2. NHIS 2023

In 2023, the NHIS conducted 29,522 Sample Adult interviews and 7,692 Sample Child interviews, achieving response rates of 47.0% and 44.9%, respectively. Data were collected through both in-person and telephone interviews, with 54.5% of interviews conducted at least partially by phone, similar to 2022 but higher than pre-pandemic levels in 2019 [36].

### 2.3. Design

NHIS is a cross-sectional, continuous survey covering the U.S. civilian noninstitutionalized population in the 50 states and the District of Columbia. Data are collected throughout the year, allowing monthly samples to be nationally representative. To manage costs and logistics, NHIS employs a geographically clustered sampling design [36].

### 2.4. Sampling

The sampling process begins by partitioning the United States into 1,689 geographic areas, based on counties or groups of counties. These areas are further stratified by population density in some states, while smaller states and the District of Columbia remain unstratified. Clusters within each stratum are selected proportionally, ensuring a representative national sample [36].

### 2.5. Sample

The NHIS targets individuals in households and noninstitutional group quarters, such as homeless shelters and group homes, but excludes active-duty military personnel, persons in long-term care institutions, and residents in correctional facilities [36].

### 2.6. Process and Interview

The U.S. Census Bureau conducts NHIS interviews under contract, deploying approximately 864 trained interviewers nationwide. Interviewers use computer-assisted personal interviewing (CAPI) technology, which facilitates question routing, real-time data entry, and data validation. Respondents receive an advance letter explaining NHIS participation, ensuring informed, voluntary consent. Interviews are conducted primarily in person, with follow-ups or special requests handled by phone [36].

### 2.7. Analytical Sample for this Paper

This paper used a sample of 22,746 participants, representative of 190,412,768 White and Black adults in the U.S. Eligibility criteria included having data on race/ethnicity, identifying as non-Latino White or non-Latino Black, and being an adult. Individuals of any other race, as well as all Latino or Hispanic individuals, were excluded from the analysis.

### 2.8. Measures

#### Educational Attainment:

Participants reported their highest level of education as one of the following categories: 00 (Never attended/kindergarten only), 01 (Grade 1–11), 02 (12th grade, no diploma), 03 (GED or equivalent), 04 (High school graduate), 05 (Some college, no degree), 06 (Associate degree: occupational, technical, or vocational program), 07 (Associate degree: academic program), 08 (Bachelor’s degree, e.g., BA, BS, BBA), 09 (Master’s degree, e.g., MA, MS, MBA), and 10 (Professional or doctoral degree, e.g., MD, JD, PhD). For analysis, we consolidated these categories into three groups: (1) Less than high school diploma (categories 0–3), (2) Some college (categories 4–7), and (3) College degree or higher (categories 8+). Educational attainment was treated as a three-level categorical variable, with “less than high school diploma” as the reference category, and “some college” and “college degree or higher” as the other two levels compared to this reference.

#### Outcomes:

Participants were asked if they had ever been told by a doctor that they have hypertension or high blood pressure.

#### Covariates:

Covariates included gender (female = 0, male = 1), age (years), marital status (other = 0, married = 1), employment status in the last week (other = 0, employed = 1), current cigarette use (no = 0, yes =1), current electronic cigarette use (no = 0, yes =1), health insurance status (insured or not), lifetime history of anxiety (no = 0, yes =1), lifetime history of depression (no = 0, yes =1), and lifetime history of high cholesterol (no = 0, yes =1). All covariates were based on self-reports.

### 2.9. Ethics

This study was conducted in compliance with ethical standards, ensuring the protection and confidentiality of participant information. All data were collected anonymously, and no identifying information was retained. The study adhered to the ethical principles outlined in the Declaration of Helsinki, emphasizing respect, beneficence, and justice in research practices. Written informed consent was obtained from all participants prior to their involvement in the study, following a clear explanation of the study’s purpose, procedures, potential risks, and benefits. Institutional Review Board (IRB) approval was obtained for NHIS to ensure that all ethical guidelines were rigorously followed throughout the research process. Current analysis used fully de-identified existing data and did not need a full IRB review.

### 2.10. Statistical Analysis

We conducted all analyses using Stata, accounting for the survey design variables, including survey weights and strata, to ensure accurate representation of the non-Latino White and non-Latino Black U.S. adult populations. Given our focus on these groups exclusively, we applied subpopulation logistic regression techniques. With four outcomes of interest, we initially ran four separate logistic regression models without interaction terms. These models tested the additive effects of race and education on each outcome, adjusting for age, gender, marital status, and employment status as covariates. As all participants were non-Latino, ethnicity was not included as a control variable. Next, we conducted a second set of four logistic regression models, this time including interaction terms between race and education. These models retained all covariates from the initial models, allowing us to assess whether the effect of education on each outcome differed between non-Latino White and non-Latino Black adults. From each logistic regression model, we reported the odds ratios (OR), standard errors (SE), 95% confidence intervals (CI), and p-values. All results presented are representative of non-Latino White and Black American adults. Minorities’ diminished returns would be inferred if: 1) the results consistently showed that higher educational attainment was inversely associated with reliance on Social Security and disability-related income (odds ratios less than one), indicating a protective effect of education; 2) the race-by-education interaction terms were significant; and 3) the interaction direction revealed that this protective effect was significantly weaker for Black adults compared to White adults. Together, these findings would suggest a diminished return on education for Black Americans in reducing reliance on Social Security and disability income sources.

## 3. Results

Table 1 shows the results of the logistic regression analysis without interaction. Regarding education, having a high school diploma was associated with a 19% reduction in odds compared to less than a high school diploma (OR = 0.81, 95% CI: 0.69–0.94, p = 0.005), and having a college education was associated with a 41% reduction in odds (OR = 0.59, 95% CI: 0.50–0.70, p < 0.001). The intercept was significant (OR = 0.01, 95% CI: 0.01–0.02, p < 0.001). Race (AIAN) was significantly associated with the outcome, with AIAN individuals having 1.52 times the odds of the outcome compared to non-AIAN individuals (OR = 1.52, 95% CI: 1.16–1.99, p = 0.002). Age was positively associated with the outcome, with each additional year increasing the odds by 5% (OR = 1.05, 95% CI: 1.05–1.06, p < 0.001). Male gender was also a significant predictor, with males having 1.48 times the odds compared to females (OR = 1.48, 95% CI: 1.37–1.60, p < 0.001). Obesity was strongly associated with the outcome, with obese individuals having 2.73 times the odds of the outcome compared to non-obese individuals (OR = 2.73, 95% CI: 2.51–2.98, p < 0.001). Lifetime anxiety was associated with higher odds of the outcome (OR = 1.40, 95% CI: 1.24–1.58, p < 0.001), as was lifetime depression, though with a smaller effect size (OR = 1.13, 95% CI: 1.00–1.27, p = 0.049). High cholesterol had a substantial positive association (OR = 2.68, 95% CI: 2.48–2.90, p < 0.001). Having health insurance was associated with higher odds of the outcome (OR = 1.41, 95% CI: 1.12–1.78, p = 0.004). Current cigarette use showed a non-significant association (OR = 1.10, 95% CI: 0.96–1.27, p = 0.174), while current e-cigarette use was also non-significant (OR = 0.98, 95% CI: 0.80–1.20, p = 0.867). Being married was not significantly associated with the outcome (OR = 1.07, 95% CI: 0.99–1.16, p = 0.105), nor was employment in the last week (OR = 0.95, 95% CI: 0.87–1.04, p = 0.288).

Table 2 shows the results of the logistic regression analysis with interaction. In terms of education, a high school diploma was associated with a 22% reduction in odds compared to less than a high school diploma (OR = 0.78, 95% CI: 0.66–0.90, p = 0.001), and a college education was associated with a 43% reduction in odds (OR = 0.57, 95% CI: 0.48– 0.68, p < 0.001). The interaction terms showed that the effect of education on the outcome differed for AIAN individuals. For AIANs with a high school diploma, the odds of the outcome were 3.12 times higher (OR = 3.12, 95% CI: 1.81–5.39, p < 0.001) than for non-AIANs with similar education levels. Similarly, for AIANs with a college education, the odds were 2.73 times higher (OR = 2.73, 95% CI: 1.25–5.95, p = 0.012) than for non-AIANs with a college education. The intercept was significant (OR = 0.01, 95% CI: 0.01–0.02, p < 0.001). Marital status (married) was not significantly associated with the outcome (OR = 1.07, 95% CI: 0.99–1.16, p = 0.099), nor was employment in the last week (OR = 0.95, 95% CI: 0.87–1.04, p = 0.287). Age showed a significant positive association with the outcome, with each additional year increasing the odds by 5% (OR = 1.05, 95% CI: 1.05–1.06, p < 0.001). Male gender was also a significant predictor, with males having 1.48 times the odds compared to females (OR = 1.48, 95% CI: 1.37–1.60, p < 0.001). Obesity was strongly associated with the outcome, with obese individuals showing 2.73 times the odds of the outcome compared to non-obese individuals (OR = 2.73, 95% CI: 2.51–2.98, p < 0.001). Current cigarette use did not show a significant association with the outcome (OR = 1.10, 95% CI: 0.96–1.26, p = 0.185), nor did current e-cigarette use (OR = 0.98, 95% CI: 0.80–1.20, p = 0.854). Lifetime anxiety was associated with higher odds of the outcome (OR = 1.40, 95% CI: 1.24–1.58, p < 0.001), and lifetime depression was marginally significant (OR = 1.13, 95% CI: 1.00–1.27, p = 0.051). High cholesterol had a strong positive association (OR = 2.68, 95% CI: 2.48–2.90, p < 0.001). Health insurance was also associated with higher odds of the outcome (OR = 1.42, 95% CI: 1.13–1.79, p = 0.003).

## 4. Discussion

This study set out with two primary aims. First, we aimed to assess the overall association between educational attainment and hypertension prevalence across all participants, hypothesizing that higher education would be associated with a lower risk of hypertension. Second, we sought to investigate whether this protective association is equally strong across racial and ethnic groups, specifically comparing AIAN adults to non-Hispanic White adults, to examine potential Minorities’ Diminished Returns (MDRs). In line with our first aim, the findings indicate that, across the overall sample, educational attainment is inversely associated with hypertension prevalence. This is consistent with a well-established body of literature showing that higher education is associated with better health outcomes [37–43], including lower hypertension risk [44–46]. The protective effect of education on hypertension can be attributed to several mechanisms: higher education often promotes health literacy, increases access to resources and preventive care, and is associated with healthier behaviors and lower levels of chronic stress. In addition, individuals with higher education levels tend to have greater employment stability, which may reduce financial strain and associated stressors that contribute to hypertension risk [37–43].

Our investigation into the second aim revealed that the protective effect of educational attainment on hypertension is significantly weaker for AIAN adults compared to their non-Hispanic White counterparts. This finding is aligned with the MDRs framework [34,47–50], which suggests that social and economic resources, such as education, do not offer the same level of health protection for minoritized groups as they do for non-Hispanic White populations. These results highlight the unique challenges that AIAN populations face in translating educational gains into health benefits and suggest that factors beyond individual education levels influence health outcomes.

The AIAN history is marked by deep-rooted trauma stemming from centuries of land dispossession, violence, and systemic oppression at the hands of European colonizers and the U.S. government [51–55]. From the earliest days of European settlement, Native lands were seized through broken treaties, forced removal, and violent displacement, often justified by a racist ideology of manifest destiny and European superiority. Mass killings and massacres, such as the Wounded Knee Massacre [56], exemplify the brutal attempts to suppress Native populations and erase their cultural heritage. This violence was compounded by policies aimed at eradicating Native traditions, language, and identity, including the forced relocation of children to boarding schools that aimed to “civilize” and assimilate them [57–60]. These acts of imperialism and systemic racism have left lasting scars on Native communities, resulting in generations of collective trauma that affects mental health, cultural continuity, and social well-being to this day. Addressing these historical injustices and recognizing their enduring impact is essential to advancing equity and healing within AIAN communities [61–63]. These adversities may result in historical trauma, which can have deteriorating health effects, regardless of individual-level exposure.

Our findings on the diminished effect of education on hypertension among AIAN adults are consistent with previous studies on MDRs, particularly those documenting weaker health returns on socioeconomic status for Black and Latino populations [64–71]. The results contribute new evidence by extending the MDRs framework to AIAN individuals, a group historically underrepresented in health disparities research. Although prior studies have documented elevated hypertension rates in AIAN populations, few have examined the extent to which education influences these rates [25,27,72].

The diminished returns of educational attainment on health outcomes for minoritized populations, including AIAN individuals, are rooted in structural inequities that limit the full benefits of socioeconomic resources. These structural causes include ongoing discrimination in healthcare, labor markets, and housing, which together restrict access to opportunities that often accompany higher education. Systemic racism perpetuates disparities in education quality, job stability, and healthcare access, leading to chronic stress and adverse health outcomes. Even when individuals from minoritized backgrounds achieve higher education levels, these structural barriers constrain their ability to leverage this achievement fully. For AIAN populations, limited healthcare infrastructure on reservations and in rural areas, coupled with discriminatory practices in predominantly non-Native healthcare settings, further undermines the health returns of education. Consequently, addressing MDRs requires not only individual-level interventions but also systemic reforms to dismantle the structural inequities that limit health benefits for minoritized groups.

The AIAN community’s experience is uniquely shaped by historical and intergenerational trauma related to land displacement, forced relocation, and cultural erasure [61,73]. The forced removal of Native peoples from their ancestral lands disrupted traditional practices, community ties, and ways of life, with profound implications for physical and mental health. This trauma is compounded by present-day issues, such as loss of access to land and resources that once supported Native communities’ self-sufficiency and cultural practices. Land loss also affects environmental quality, food access, and economic stability, factors that collectively impact health outcomes, including hypertension. The continued connection to land remains central to AIAN identity, health, and well-being, and the unique historical trauma linked to land displacement must be considered when examining the health of AIAN individuals. Recognizing these complex sociohistorical factors is essential for creating culturally sensitive policies and interventions that address the unique health needs of AIAN communities [74–77].

The observed diminished protective effect of education for AIAN adults could be attributed to a range of structural and social factors. Chronic exposure to discrimination, limited access to high-quality healthcare, and historical inequities may all undermine the health benefits that are typically associated with higher education. Additionally, labor market discrimination and geographic limitations may restrict AIAN individuals’ economic opportunities, even with higher educational credentials, further eroding the potential health benefits associated with educational attainment.

### 4.1. Implications

These findings underscore the need for public health interventions and policies that consider the unique contexts of AIAN communities. Enhancing health literacy and access to preventive healthcare in AIAN communities, coupled with policies aimed at reducing labor market discrimination, may improve health outcomes and maximize the health benefits of educational attainment in these populations. Addressing the broader social and environmental determinants of health for AIAN populations is crucial to reducing hypertension disparities and advancing health equity.

### 4.2. Limitations

While this study provides valuable insights, certain limitations should be noted. The reliance on self-reported hypertension diagnoses may introduce reporting biases. Additionally, while we adjusted for several sociodemographic variables, unmeasured factors such as chronic stress and healthcare access could also influence our results. Future research should explore MDRs across a broader range of health outcomes and use longitudinal data to better understand the mechanisms underlying these disparities.

## 5. Conclusion

This study offers evidence that educational attainment, while generally protective against hypertension, provides limited health benefits for AIAN adults compared to non-Hispanic Whites. Our findings highlight the importance of addressing structural barriers that hinder AIAN communities from fully realizing the health advantages associated with education. To reduce hypertension disparities, public health efforts must address both individual and systemic factors affecting AIAN populations.

## Figures and Tables

**Table 1.** Logistic regression without interaction

	OR	SE	95%	CI	p
Race (AIAN)	1.52	0.21	1.16	1.99	0.002
Age (Years)	1.05	0.00	1.05	1.06	< 0.001
Gender (Male)	1.48	0.06	1.37	1.60	< 0.001
Obese	2.73	0.12	2.51	2.98	< 0.001
Current Cigarette Use	1.10	0.08	0.96	1.27	0.174
Current E-Cigarette Use	0.98	0.10	0.80	1.20	0.867
Anxiety (Lifetime)	1.40	0.09	1.24	1.58	< 0.001
Depression (Lifetime)	1.13	0.07	1.00	1.27	0.049
High Cholesterol (Lifetime)	2.68	0.11	2.48	2.90	< 0.001
Health insurance (Insured)	1.41	0.17	1.12	1.78	0.004
Married	1.07	0.05	0.99	1.16	0.105
Employed (Last Week)	0.95	0.04	0.87	1.04	0.288
Education					
Less Than High School Diploma					
High School Diploma	0.81	0.06	0.69	0.94	0.005
College	0.59	0.05	0.50	0.70	< 0.001
Intercept	0.01	0.00	0.01	0.02	< 0.001

Note: American Indian and Alaska Native (AIAN)

**Table 2.** Logistic regression without interaction

	OR	SE	95%	CI	p
Race (AIAN)	0.59	0.14	0.38	0.93	0.022
Age (Years)	1.05	0.00	1.05	1.06	< 0.001
Gender (Male)	1.48	0.06	1.37	1.60	< 0.001
Obese	2.73	0.12	2.51	2.98	< 0.001
Current Cigarette Use	1.10	0.08	0.96	1.26	0.185
Current E-Cigarette Use	0.98	0.10	0.80	1.20	0.854
Anxiety (Lifetime)	1.40	0.09	1.24	1.58	< 0.001
Depression (Lifetime)	1.13	0.07	1.00	1.27	0.051
High Cholesterol (Lifetime)	2.68	0.11	2.48	2.90	< 0.001
Health insurance (Insured)	1.42	0.17	1.13	1.79	0.003
Married	1.07	0.05	0.99	1.16	0.099
Employed (Last Week)	0.95	0.04	0.87	1.04	0.287
Education					
Less Than High School Diploma					
High School Diploma	0.78	0.06	0.66	0.90	0.001
College	0.57	0.05	0.48	0.68	< 0.001
AIAN x High School Diploma	3.12	0.87	1.81	5.39	< 0.001
AIAN x College	2.73	1.08	1.25	5.95	0.012
Intercept	0.01	0.00	0.01	0.02	< 0.001

Note: American Indian and Alaska Native (AIAN)
